# Individual *C. elegans* neurons display differential sensitivity to *smn-1* silencing

**DOI:** 10.26508/lsa.202603647

**Published:** 2026-07-13

**Authors:** Sara Savaheli, Ivan Gallotta, Pamela Santonicola, Federica Cieri, Simon Berger, Delphine Dargere, Elia Di Schiavi, Denis Dupuy

**Affiliations:** 1 CNRS, INSERM, UMR 5320, U1212, University of Bordeaux , Bordeaux, France; 2 Institute of Biosciences and BioResources (IBBR), National Research Council (CNR) , Naples, Italy; 3 Institute of Genetics and Biophysics “Adriano Buzzati-Traverso” (IGB-ABT), National Research Council (CNR) , Naples, Italy; 4 Department for Molecular Life Sciences, University Zurich , Zurich, Switzerland

## Abstract

Spinal muscular atrophy is a neuromuscular disorder primarily caused by mutations in the *SMN1* (Survival of Motor Neuron 1) gene. *SMN1* is ubiquitously expressed and encodes a protein essential for the assembly of small nuclear ribonucleoproteins, key components of pre-mRNA splicing. The SMN protein also participates in several other fundamental cellular processes, including RNA transport, regulation of actin dynamics, transcription, and translation. While multiple hypotheses have been put forward to explain the selective motor neurons (MNs) vulnerability to SMN deficiency, the precise mechanisms involved remain incompletely understood. In this study, we used neuron-specific *smn-1* RNAi silencing in D-type MNs or in touch receptor neurons in *C. elegans*. In touch receptor neurons, *smn-1* silencing caused distinct defects in neuronal process morphology. Our results reveal pronounced neuron-specific differences in sensitivity within the neurons of *C. elegans*, providing a robust framework to dissect the mechanisms underlying selective neuronal vulnerability of spinal cord MNs in spinal muscular atrophy.

## Abstract (precis)

Cell specific *smn-1* silencing in *C. elegans* caused distinct defects in morphology with pronounced neuron-specific differences in sensitivity between neurons of the same class. This provides a robust framework to dissect the mechanisms underlying selective neuronal vulnerability of SMA.

## Introduction

Spinal muscular atrophy (SMA) is a genetic neuromuscular disorder first described by Guido Werdnig in 1891 ((Werdnig, 1891, according to Mercuri (2021)). SMA is characterized by the loss of motor neurons (MNs) in the anterior horn of the spinal cord and mutations of the Survival of Motor Neuron 1 (*SMN1*) gene account for over 95% of SMA cases (Crawford & Pardo, 1996; Butchbach, 2016). SMN protein, the product of the *SMN1* gene, is best known for its role in snRNP assembly for pre-mRNA splicing (Pellizzoni et al, 2002), but it also plays other functions, including RNA transport, cytoskeletal regulation, transcription, and translation (Singh et al, 2017). The SMN protein is ubiquitously expressed, yet its deficiency predominantly affects MNs in the spinal cord. Although SMA has more recently been recognized as a multisystem disorder involving functional impairments in multiple neuronal and non-neuronal tissues (Abati et al, 2020; Yeo & Darras, 2021), MNs of spinal cord remain disproportionately vulnerable and represent the most prominent feature. This neuronal vulnerability has prompted extensive investigation into why these cells are mainly affected (Ruggiu et al, 2012; Simon et al, 2017). Increasing evidence from both human and animal studies now suggests that subtle developmental abnormalities may predispose MNs to later degeneration (Crawford & Pardo, 1996; McGovern et al, 2008; Martínez-Hernández et al, 2013; Kong et al, 2021). In addition, the study by Irene Faravelli demonstrated dysregulated neuronal differentiation programs, supporting the need for intervention during the optimal developmental window to maximize therapeutic efficacy (Faravelli et al, 2025). Building on this emerging view, we aim to further investigate selective vulnerability of different types of neurons to *smn-1* gene perturbation using *Caenorhabditis elegans* as an in vivo model.

*C. elegans* is widely used in biological research because it is highly amenable to genetic manipulation, possesses a simple and well-characterized nervous system, and has a fully mapped connectome, representing all neural connections (White et al, 1986; Hall & Russell, 1991). These features make it an advantageous system for dissecting intrinsic, cell-autonomous neuronal differences associated with SMN loss. The *C. elegans* nervous system develops in two distinct developmental waves: of the 302 neurons that constitute the *C. elegans* hermaphrodite adult nervous system, 222 arise during embryogenesis, and the remaining are born post-embryonically during the L1 stage (Sulston et al, 1983). The GABAergic inhibitory D-type MNs form a major subgroup, further divided into six Dorsal D-type (DD) and 13 Ventral D-type (VD) neurons. DD neurons appear during embryogenesis, while VD neurons appear at the end of the first larval stage (Sulston et al, 1983; White et al, 1986). These inhibitory MNs coordinate the alternating contraction of the dorsal and ventral longitudinal muscles, producing the animal’s characteristic sinusoidal movement. When the dorsal muscles contract, VD neurons inhibit the ventral muscles to allow relaxation; conversely, when the ventral muscles contract, DD neurons inhibit the dorsal muscles. This reciprocal pattern of activation and inhibition generates the smooth sinusoidal waves that drive forward locomotion (Howell et al, 2015; Zhen & Samuel, 2015).

Touch receptor neurons (TRNs) are a group of glutamatergic mechanosensory neurons specialized for the perception of gentle touch, which include the anterior lateral microtubule cells (ALMs), posterior lateral microtubule cells (PLMs), the anterior ventral microtubule cell (AVM), and the posterior ventral microtubule cell (PVM) (Chalfie and Sulston, 1981; Pereira et al, 2015). Like the D-type MNs, TRNs are generated in two distinct developmental periods: the ALM and PLM neurons arise during embryogenesis, whereas the AVM and PVM neurons are born during the first larval stage (Sulston et al, 1983).

In *C. elegans*, systemic RNAi against *smn-1* or deletion of *smn-1* results in larval lethality. Conversely, available hypomorphic models do not always exhibit obvious neuronal loss, limiting the ability to investigate the degenerative process directly (Miguel-Aliaga et al, 1999; Briese et al, 2009; Doyle et al, 2020). To overcome this, we used neuron-specific RNAi constructs (Esposito et al, 2007) to selectively silence *smn-1* in defined neuronal populations, yielding viable animals in which degeneration of targeted neurons can be studied. The RNAi constructs were driven by two distinct neuron-specific promoters – the *unc-25 Short* promoter (*Punc-25S*) for D-type MNs and the *mec-*3 promoter (*Pmec-3*) for TRNs and RNAi is activated only in the targeted neurons when terminal differentiation triggers the activation of the selected promoter (Gallotta et al, 2016). In Gallotta et al (2016), the silencing of *smn-1* in MNs caused a cell-autonomous, age-dependent degeneration of this class of neurons detected as locomotory defects and the disappearance of presynaptic and cytoplasmic fluorescent markers in targeted cells. The confirmed a neuronal death of MNs as revealed by positive reactivity to genetic and chemical cell-death markers. We also showed that genes of the classical apoptosis pathway are involved in the *smn-1*-mediated neuronal death, and that this phenotype can be rescued by the expression of human *SMN1*, indicating a functional conservation between the two orthologs. In the present work, we extended the analysis to the two subclasses of MNs, the DD and VD types, and to another neuronal class. To visualize the phenotypic consequences of *smn-1* dysregulation, we labeled the targeted neurons by expressing fluorescent reporters driven by distinct neuron-specific promoters. Neuronal morphology then assessed from the first larval stage through day 4 of adulthood corresponding to ∼7 d post-egg under standard conditions.

## Results

### Heterogeneous neuron-specific response to *smn-1* RNAi in GABAergic MNs

**Figure 1.**
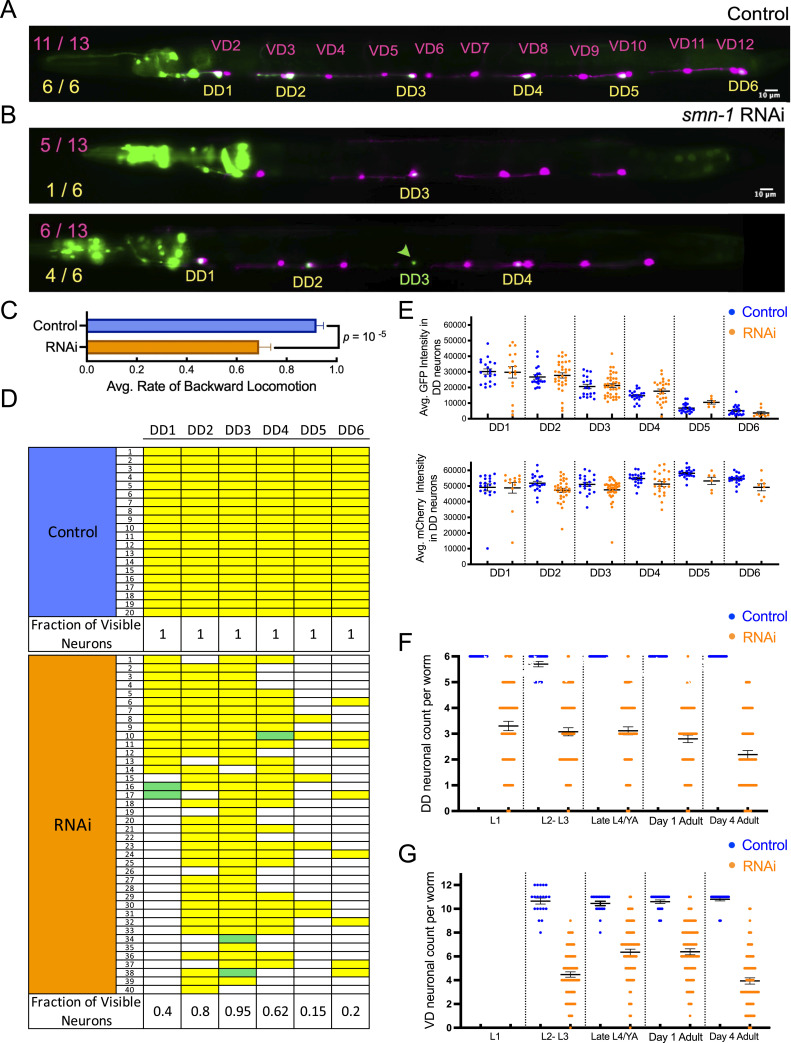
Differential effect of *smn-1* silencing on VD and DD Neurons. **(A)** Representative image of VD and DD motor neurons in the control reporter strain (DUD2204) at the late L4/young adult stage carrying *Pflp-13*::GFP (expressed in DD neurons) and *Pttr-39*::mCherry (expressed in VD and DD neurons). Eleven out of thirteen VD neurons (magenta) and all six DD neurons (white) are visible. Head neurons fluorescence corresponds to the co-injection marker *Pttx-3::GFP.* **(B)** Representative images of two animals (strain DUD2205) at the late L4/young adult stage, carrying *Punc-25S::smn-1* RNAi *(smn-1* silencing), *Pflp-13*::GFP (GFP expressed in DD neurons) and *Pttr-39*::mCherry (mCherry expressed in VD and DD neurons). Upper image: five out of thirteen VD neurons (magenta) and only one DD neuron (white) is visible. Lower image: six out of thirteen VD neurons (magenta) and four out of six DD neurons (white) are visible. DD3 neuron (green arrowhead) expresses only GFP instead of the expected co-expression of GFP and mCherry. Head neurons fluorescence corresponds to the co-injection markers *Pttx-3::*GFP and *Pchs-2::*GFP. **(C)** Backward locomotion assay at day 2 adult stage in 100 controls and 100 *smn-1*-silenced worms. Backward movement was assessed following mechanical stimulation. Differences between groups were analyzed using Fisher’s exact test. **(D)** Heat map showing the presence or absence of DD neurons in individual worms. Each row represents a single animal. A total of 20 control worms (upper panel) and 40 *smn-1*–silenced worms (lower panel) at L4 larval stage were analyzed. The fraction of visible fluorescent neurons for each DD neuron, relative to the total number of worms examined, is indicated below the heat map. Yellow cells represent a visible neuron expressing GFP and mCherry, green in cells indicate neuron expressing only GFP, white cells indicate non visible fluorescence. **(E)** Comparative analysis of *Pflp-13*::GFP (upper plot) and *Pttr-39*::mCherry (lower plot) reporter expression by measuring fluorescence intensity (arbitrary units) in each visible DD neuron at late L4/young adult stage in the control (blue dots) and in *smn-1*–silenced strains (orange dots). n = 20 control worms and n = 40 *smn-1*–silenced worms at L4 larval stage. **(F, G)** Comparative analysis of motor neuron number in control and *smn-1*–silenced strains by counting visible DD neurons (F) and VD neurons (G) in the control (blue dots) and in *smn-1*–silenced strains (orange dots) at different time points. n = 20 control worms and n = 80 *smn-1*–silenced worms. VD, Ventral D-type; DD, Dorsal D-type; SMN, survival of motor neuron. Source data are available for this figure.

**Figure S1.**
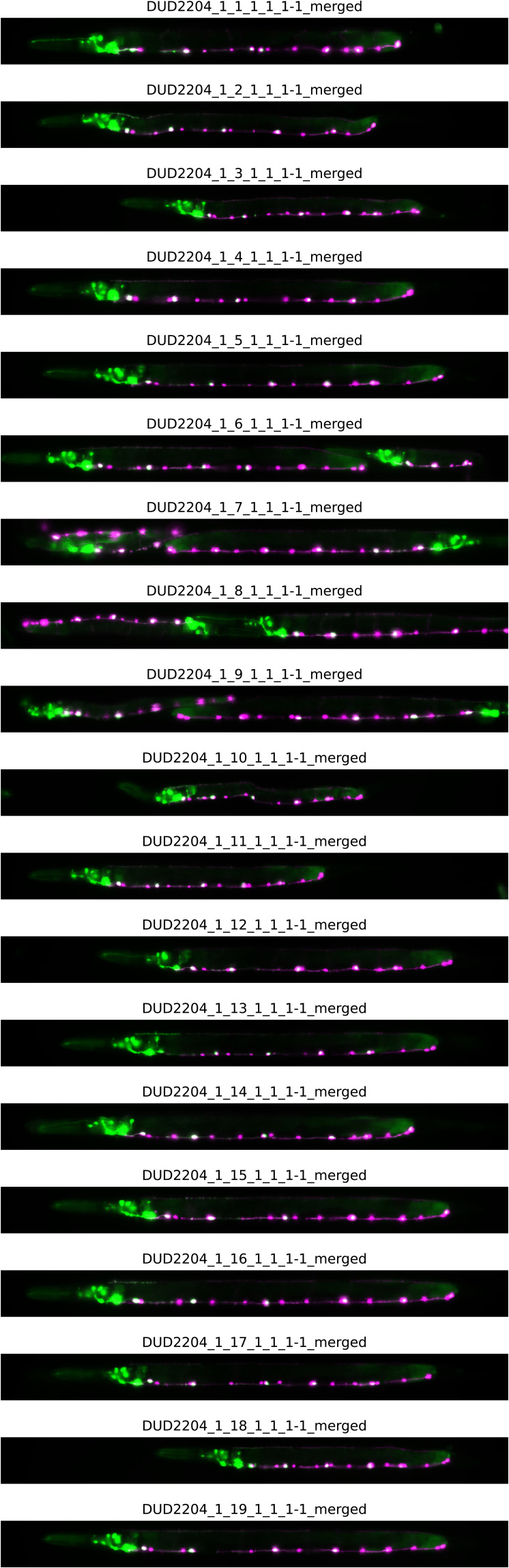
Expression of the fluorescent reporters at the late L4/young adult stage. Representative fluorescence images of the of VD and DD motor neurons in the control reporter strain (DUD2204) at the late L4/young adult stage carrying *Pflp-13*::GFP (expressed in DD neurons) and *Pttr-39*::mCherry (expressed in VD and DD neurons). Head neurons fluorescence corresponds to the co-injection marker *Pttx-3*::GFP.

**Figure S2.**
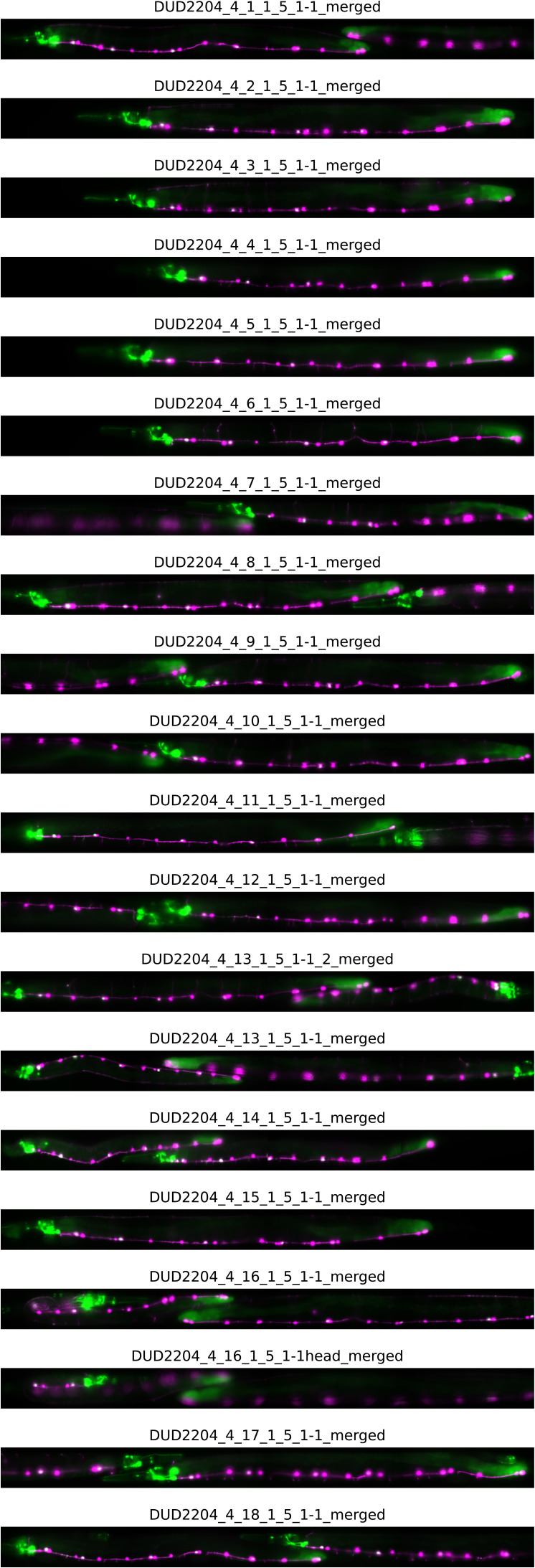
Expression of the fluorescent reporters at Day 1 adult stage. Representative fluorescence images of the of VD and DD motor neurons in the control reporter strain (DUD2204) at Day 1 adult stage carrying *Pflp-13*::GFP (expressed in DD neurons) and *Pttr-39*::mCherry (expressed in VD and DD neurons). Head neurons fluorescence corresponds to the co-injection marker *Pttx-3*::GFP.

**Figure S3.**
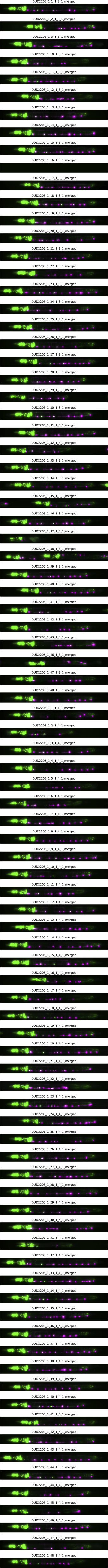
Effect of *smn-1* silencing on VD and DD Neurons at the late L4/young adult stage. Representative images of *smn-1* silenced strain (DUD2205) at the late L4/young adult stage, carrying *Punc-25S*::smn-1 RNAi (smn-1 silencing), *Pflp-13*::GFP (GFP expressed in DD neurons) and *Pttr-39*::mCherry (mCherry expressed in VD and DD neurons). Head neurons fluorescence corresponds to the co-injection markers *Pttx-3*::GFP and *Pchs-2*::GFP.

**Figure S4.**
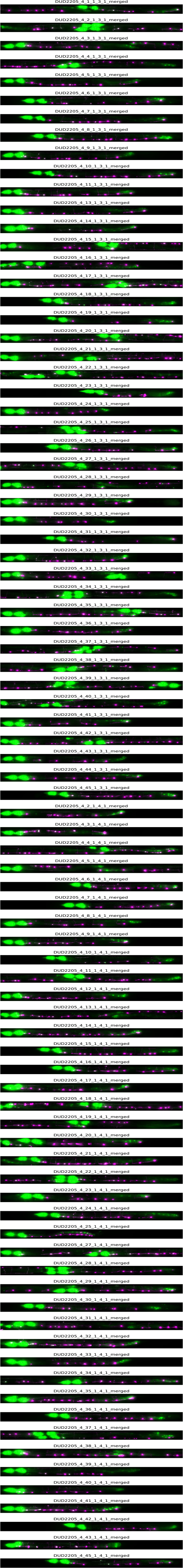
Effect of *smn-1* silencing on VD and DD Neurons at Day 1 adult stage. Representative images of *smn-1* silenced strain (DUD2205) at Day 1 adult stage, carrying *Punc-25S*::smn-1 RNAi (*smn-1* silencing), *Pflp-13*::GFP (GFP expressed in DD neurons) and *Pttr-39*::mCherry (mCherry expressed in VD and DD neurons). Head neurons fluorescence corresponds to the co-injection markers *Pttx-3*::GFP and *Pchs-2*::GFP.

We previously assessed the impact of *smn-1* silencing on MN survival using neurons-specific RNAi, with neurons visualized via a reporter strain in which the *unc-47* promoter drives GFP expression in all D-type MNs (Gallotta et al, 2016). In the present study, we used dual labeling to differentiate the effect of *smn-1* silencing on ventral and dorsal D-type MNs (VD and DD neurons). To this end, we used the *Pttr-39*, driving mCherry expression in both VD and DD neurons (Jospin et al, 2009), and the *Pflp-13*, to drive GFP expression only in DD neurons (Thompson-Peer et al, 2012). As a result, VD neurons were labeled exclusively with mCherry (magenta neurons in Fig 1A and B), whereas DD neurons co-expressed both mCherry and GFP (white neurons in Fig 1A and B). We confirmed a significant reduction in the total number of visible D-type MNs in the *smn-1*–silenced strain compared with the control strain at the late L4/young adult stage and Day 1 adult stage (Figs 1A and B, S1, S2, S3, and S4). We also confirmed a significant reduction of backward locomotion following light touch behind the pharynx in RNAi animal compared with control (Fig 1C, as previously described in Gallotta et al [2016]).

Individual DD neurons were identified based on fluorescent markers, neuronal process, and anatomical position in 20 control and 40 *smn-1*–silenced animals at the late L4/young adult stage. While all 6 DD neurons were always visible in the control strain, we observed neuron-specific depletion in the RNA-interfered strain. DD5 and DD6 were the most sensitive to *smn-1* gene perturbation, with only 1–2 animals out of 10 displaying detectable fluorescence in these neurons. DD1 was the next most affected neuron, visible in only 4 out of 10 animals. DD2 and DD3 were the most resilient ones, detected in 8 to 9 RNAi animals out of 10, respectively (Fig 1D). In addition, we observed several DD neurons that only express GFP instead of both expected fluorescent reporters (green arrowhead in Fig 1B and green cells in Fig 1D and Video 1).

Video 1 Long-term time-lapse imaging of D-type motor neurons in *smn-1*–silenced worms to monitor the birth of VD neurons (red neurons). During the L1-to-L2 transition, 4 out of 13 VD neurons in the complete set appeared. In this video, the DD3 neuron is visible expressing GFP alone, with no co-expression of mCherry. Time-lapse images were captured at 15-min intervals. Download video

Measuring the fluorescence intensity revealed that individual neurons had consistently different fluorescence level across individual animals. For example, in DD neurons there is an anteroposterior gradient for *Pflp-13* driven GFP-intensity observable both in control and *smn-1*–silenced worms (Fig 1E, upper panel). By contrast, in *Pttr-39::*mCherry expressing animals, the reporter levels appeared relatively uniform across DD neurons, with no pronounced intra-subclass differences (Fig 1E, lower panel). Overall, the fluorescence levels of both reporters are not significantly affected by *smn-1* silencing compared with controls (Fig 1E).

**Figure S5.**
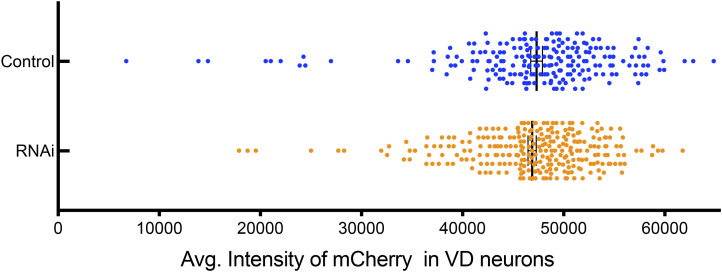
Effect of smn-1 silencing on VD neurons reporter expression. Comparative analysis of *Pttr-39*::mCherry reporter intensity in VD neurons at late L4/young adult stage in the control (blue dots) and in *smn-1*–silenced strains (orange dots).

DD and VD neurons were counted at the L1, L2/L3, late L4/young adult stages, as well as at day 1 and day 4 of adulthood. DD neuron numbers were already reduced at the L1 stage in *smn-1*–silenced animals compared with controls and exhibiting a slight decrease in number during early adulthood (Fig 1F). The VD neurons appear during the L2 stage, since, as expected they were absent at L1 stage in both control and *smn-1*–silenced strains. By the L2 stage, when the full complement of VD neurons is visible in control worms, *smn-1*–silenced animals display a marked reduction, indicating a developmental deficit. We observed a slight increase in VD counts from L2 to late L4/young adult stages, followed by a progressive decline by day 4 of adulthood (Fig 1G). Measuring the fluorescence intensity, fluorescence levels of *Pttr-39::*mCherry reporter are not significantly affected by *smn-1* silencing compared with controls (Fig S5).

We used long-term time-lapse imaging (Berger et al, 2021) to monitor VD neurons during the transition from L1 to L2 in *smn-1*–silenced animals and confirmed that some VD neurons failed to appear in this period (see Video 1 and Video 2).

Video 2 Long-term time-lapse imaging of D-type motor neurons in *smn-1*–silenced worms to monitor the birth of VD neurons (red neurons). During the L1-to-L2 transition, 9 out of 13 VD neurons in the complete set appeared. Time-lapse images were captured at 15-min intervals. Download video

### Heterogeneous response to TRNs-specific *smn-1* RNAi

**Figure 2.**
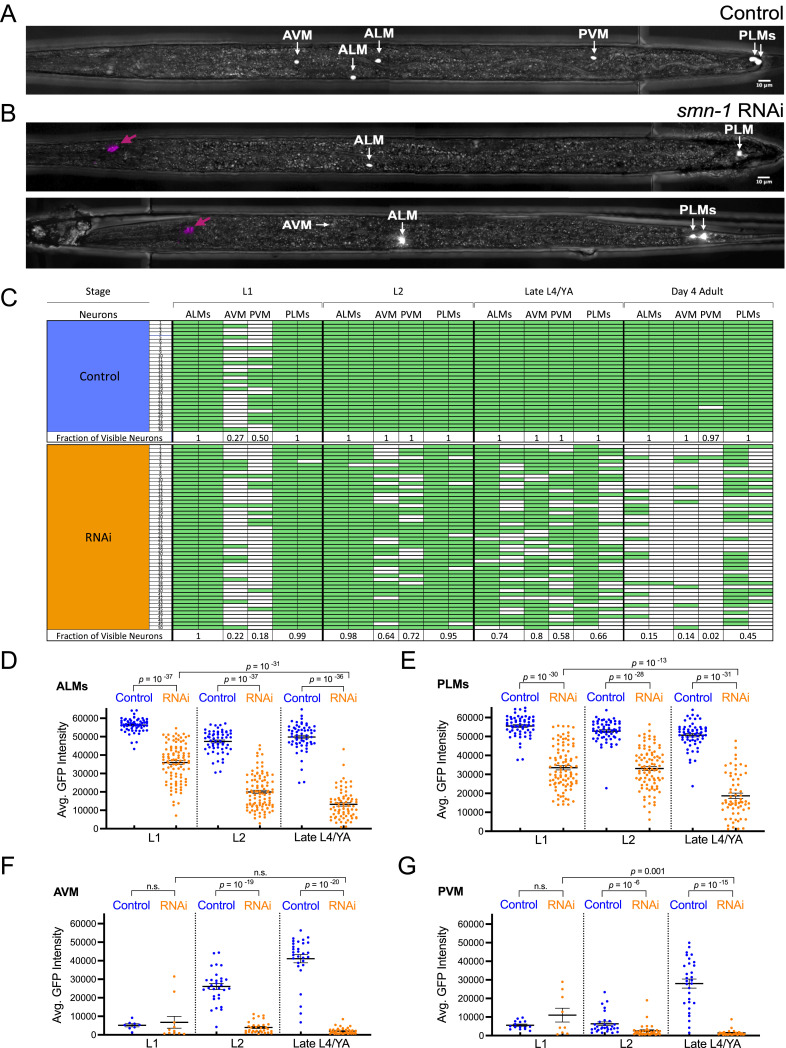
Distinct effects of *smn-1* silencing on TRNs neuronal development and survival. **(A)** Representative images of TRNs in the control strain carrying *Pmec-4*::GFP reporter at the late L4/young adult stage stage. All six TRNs are visible, as indicated by the white arrows. **(B)** Representative images of two *smn-1*–silenced animals (strain NA2397) carrying *Pmec-4::*GFP and *Pmec-3S::smn-1* RNAi transgenes, at the late L4/young adult stage stage. Visible TRNs are indicated by white arrows. The number of visible TRNs is reduced to 2 in the upper image and 4 in the lower image. The magenta arrow indicates the co-injection marker. **(C)** Heat map showing the presence or absence of TRNs in individual worms. Each row represents a single animal. A total of 30 control worms (upper panel) and 50 *smn-1*–silenced worms (lower panel) at four different time points were analyzed. The proportion of missing neurons for each TRNs, relative to the total number of worms examined, is indicated below the heat map. A green cell represents a visible neuron expressing *Pmec-4::*GFP, empty cell means non visible neuron. **(D, E, F, G)** Quantification of the expression of *Pmec-4*::GFP reporter (control: blue dots; *smn-1*–silenced: orange dots) by measuring fluorescence intensity (arbitrary units) at three different timepoints (L1, L2, and late L4/young adult) in the four classes of neurons: ALMs, PLMs, AVM and PVM. When significant, exact *P*-values are displayed above the bracket. A total of 30 control worms and 50 *smn-1*–silenced worms at three different time points were analyzed. AVM, anterior ventral microtubule cell; ALM, anterior lateral microtubule cell; PLM, posterior lateral microtubule cells; PVM, posterior ventral microtubule cell; SMN, survival of motor neuron. Source data are available for this figure.

For visualization of the TRNs, we used a different transgene that also enables clear visualization of neurons structure and morphology: GFP driven by the *mec-4* promoter (*Pmec-4*) (Fig 2A). We crossed the TRN reporter strain with strain *dudIs2202* in which *smn-1* is selectively silenced in TRNs and observed a marked reduction of visible fluorescent neurons (Fig 2B).

Quantification of individual TRNs in the *smn-1*–silenced strain revealed that, although the embryonically derived ALMs and PLMs neurons were visible at the L1 stage in comparable level to the control, their numbers progressively declined over time. By the day 4 of adult stage, the proportion of visible neurons decreased to 0.15 in the ALMs and 0.45 in the PLMs (Fig 2C). For post-embryonically born neurons (AVM and PVM), in about half of the animals we did not detect either or both AVM and PVM by the L2 stage. From L2 to L4 stage the fraction of visible AVMs went from 0.64 to 0.8, potentially indicating a developmental delay. For PVM in contrast, this fraction went from 0.72 to 0.58. By day 4 of adult stage, the ratio of missing neurons rose to 0.86 in AVM and 0.98 in PVM (Fig 2C).

GFP intensity was markedly lower in *smn-1* silenced worms than in the control in all TRNs. This reduction became more pronounced over time and was accompanied by a progressive decrease in the number of detectable neurons (Fig 2D–G). Overall, our observations indicate that *smn-1* silencing affects both the development and survival of TRNs in a neuron-specific manner.

### Structural abnormalities in ALM and PLM neuronal processes under *smn-1* silencing

**Figure S6.**
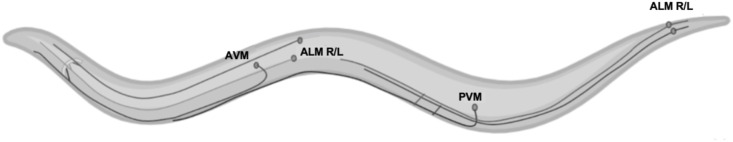
Anatomy of ALM and PLM neurite process. ALM (ALML, ALMR) neurons are located in the midbody and extend a long anterior process toward the pharynx, with a branch to the nerve ring. PLM (PLML, PLMR) neurons are located in the lumbar ganglion and extend a short posterior process and a long anterior process that ends near the ALM. AVM and PVM are positioned in the ventral body and extend a single long process along the ventral nerve cord. AVM, anterior ventral microtubule cell; ALM, anterior lateral microtubule cell; PLM, posterior lateral microtubule cells.

**Figure 3.**
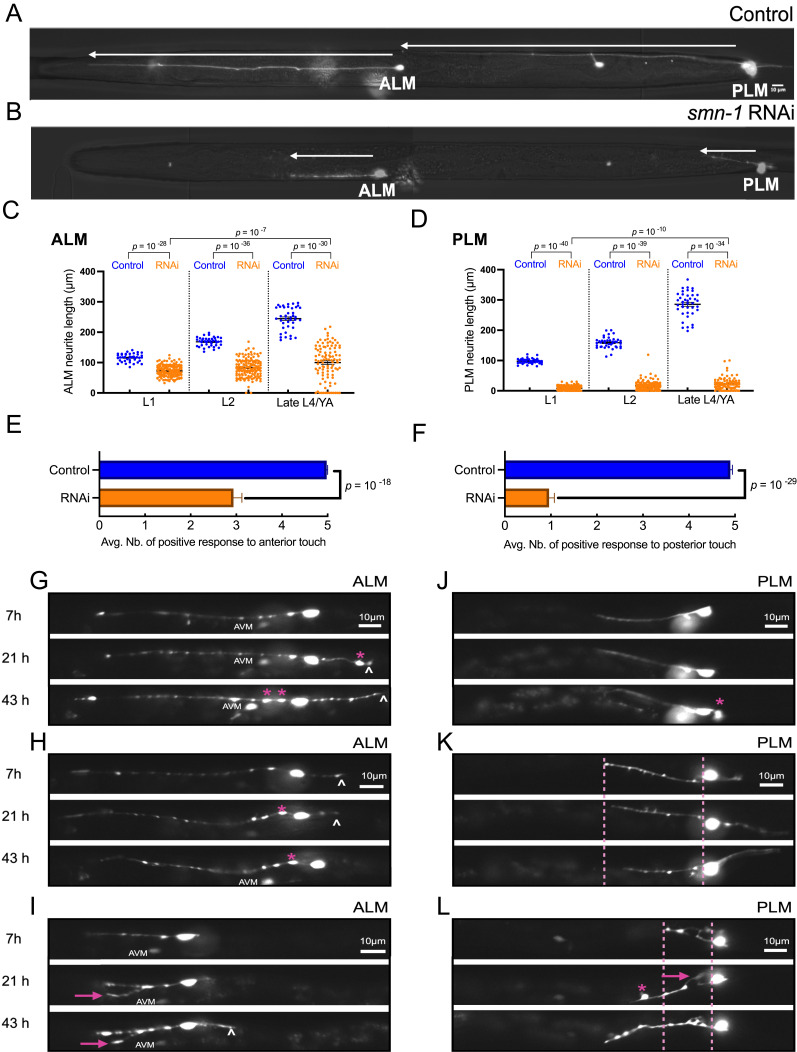
Degeneration of ALM and PLM neurites in *smn-1*–silenced strain. **(A)** Representative image of the neuronal processes of ALMs and PLMs in the control strain carrying *Pmec-4::*GFP at the late L4/young adult stage. **(B)** Representative image of neuronal processes of ALMs and PLMs in the *smn-1*–silenced strain carrying *Pmec-4::*GFP and *Pmec-3S::smn-1* RNAi at the the late L4/young adult stage. **(C, D)** Quantification of ALM and PLM neurite lengths (μm) was performed across different developmental stages. Comparative analysis of ALM (C) and PLM (D) neuronal process length in control (blue dots) and *smn-1*–silenced strains (orange dots). A total of 20 control worms and 70 *smn-1*–silenced worms were examined in this study. Significance of neurite length distributions difference between conditions were statistically evaluated. When significant, exact *P-*values are displayed above the horizontal bracket. **(E, F)** Light-touch assay in 50 controls and 50 *smn-1*-silenced worms at young adult stage. Light-touch assay was assessed following 5 mechanical stimulations in head (anterior) and tail (posterior) of worms. Differences between groups were analyzed using the Mann–Whitney test. **(G, H, I, J, K, L)** Representative image of neuronal process abnormalities in *smn-1*–silenced strain. Ectopic posterior neurite elongation in ALM neuron (white arrowhead) (G, H, I). Bulging in the neuronal process (magenta star) of ALM (G, H) and PLM (J, L). Abnormally branched neurites (magenta arrow) in ALM (I) and PLM (L). Progressive shortening of the anterior neurite in PLM (K) and abnormal dynamic of the anterior neurite in PLM (L). ALM, anterior lateral microtubule cell; PLM, posterior lateral microtubule cells; SMN, survival of motor neuron. Source data are available for this figure.

ALM and PLM provide an ideal system for studying morphological defects in the neurite process. ALM neurons are positioned in the midbody and extend a long anterior process that branches ventrally into the nerve ring (see Fig S6). In a subset of animals, ALM neurons also exhibit a short posterior protrusion extending less than twice the length of the cell body. PLM neurons on the other hand are located in the lumbar ganglion, extending a long anterior process that terminates near the ALM cell body, and a short posterior process (Fig 3A; Chalfie et al, 1985; Kirszenblat et al, 2013). We quantified the size of the anterior process in ALM and PLM neurons after hatching, at L1, L2, and late L4/young adult stages in control and *smn-1*–silenced animals (Fig 3A and B).

At the L1 stage, ALM and PLM anterior processes in nearly all *smn-1*–silenced worms are markedly shorter than those in control worms (Fig 3C and D). Throughout development, while the ALM neurite process in control animals grows steadily, in *smn-1*–silenced worms they remained markedly short (Fig 3B–D). At the late L4/young adult stage, we also observed ALM neurons lacking a neurite, indicating not only a failure of neurite extension but also suggesting that some initially formed neurites undergo retrograde degeneration (Fig 3C). In the PLM neurons, instead, there was no measurable growth of the neurite over time (Fig 3D), once again uncovering distinct phenotypes between different neurons. We assessed functional changes resulting from neuronal process defects by performing a light-touch assay in controls and *smn-1*–silenced worms. We observed a significant reduction of light touch response in young adult animals in *smn-1*–silenced worms, which was more pronounced in posterior touch, consistent with the more severe neuronal process defects observed in PLMs (Fig 3E and F).

We studied neuronal process through long-term time-lapse imaging experiments starting from mid-L2 stage for 48 h (Berger et al, 2021). We consistently observed short anterior processes across all examined neurons and some becoming progressively shorter before being eventually lost, followed by disappearance of the corresponding neuronal cell body, indicating that neurite disappearance precedes neuronal loss (Fig 3K and Video 3).

Video 3 Long-term time-lapse imaging of PLMs in *smn-1*–silenced worms to monitor the survival of TRNs during larval stage. At the beginning of the video, both PLM neurons are visible (two neurons in the right end of video). From ∼8 h onward, one PLM neuron disappears, leaving only a single PLM neuron visible. Time-lapse images were captured at 15-min intervals. Download video

Neurite branching was also detected only in a subset of these shortened processes (Fig 3I, ALM; Fig 3L, PLM). In addition, we observed elongated ectopic posterior neurites in certain ALM neurons that progressively extended over time (Fig 3G and I), whereas in others, existing ectopic neurites gradually shortened and eventually disappeared (Fig 3H).

Another notable pattern is the presence of focal swellings and bulges along the length of the neuronal processes in *smn-1*–silenced worms (Fig 3G, H, J, and L). These swellings mildly increased in size over time.

Overall, our findings showed the presence of neuronal process defects preceding neuronal cell body disappearance.

## Discussion

SMN is a multifunctional protein with essential roles in RNA processing and additional roles in diverse cellular processes (Singh et al, 2017). SMN is broadly expressed across tissues, and while severe SMA can affect multiple cell types, MNs appear to be more vulnerable to SMN depletion (Anagnostou et al, 2005; Jablonka et al, 2006; Abati et al, 2020; Yeo & Darras, 2021). Several studies have investigated the mechanisms underlying this selective vulnerability because of understanding these mechanisms could guide the development of complementary and more targeted therapeutic strategies (Ruggiu et al, 2012; Simon et al, 2017). In this study, we took advantage of the simple and fully mapped nervous system of *C. elegans* to track SMA-associated neuronal alterations. Although several SMA models have been developed in *C. elegans*, the study of SMA at later developmental stages remains limited due to the larval lethality of complete *smn-1* knockout (*smn-1*(*ok355*) mutant) and of systemic RNAi of *smn-1*. In addition, hypomorphic models (e.g., *smn-1*(*cb131*) and *smn-1*(*gk118916*) mutants) do not exhibit obvious neuronal loss at larval and adult stages, limiting their ability to recapitulate the neurodegenerative features observed in SMA (Miguel-Aliaga et al, 1999; Briese et al, 2009; Doyle et al, 2020). Interestingly, *smn-1*(*gk118916*) show D-type MNs degeneration at 9 d of adulthood, but, being a genetic mutant, it does not allow to assess the cell-autonomous role played by *smn-1* in specific subclasses of neurons. To overcome this, we used a model in which *smn-1* is silenced in defined neuronal populations using neuron-specific RNAi approach (Esposito et al, 2007). Thus, worms remain viable and healthy, and neurons develop with normal levels of SMN until *smn-1* silencing onset once the promoter driving RNAi expression becomes active. Using this strategy, earlier studies have shown that *smn-1* silencing in *C. elegans* GABAergic MNs significantly compromise the survival of neurons in an age-dependent manner (Gallotta et al, 2016). Here, we show that *smn-1* silencing differentially affects the two GABAergic MNs subtypes: VD and DD (Fig 1F and G). Notably, DD neurons display distinct sensitivities to *smn-1* loss, indicating intrinsic differences between each individual DD neuron (Fig 1D). These differences are also visible in the distinct level of reporter expression we observed (Fig 1E). In addition, we observed that *smn-1* perturbation also affects the development of VD neurons, with an initial delay in the appearance of a subset of these neurons, followed by the previously described degeneration (Gallotta et al, 2016). We found that, VD neurons exhibit a more pronounced progressive loss compared with DD neurons (Fig 1G). Together, these findings indicate distinct vulnerability profiles among MN subtypes and between individual cells with those subtypes.

We next examined mechanosensory neurons and observed comparable defects in TRNs, which was not unexpected, given that sensory neurons also become compromised in more severe cases in human and animal studies (Anagnostou et al, 2005; Jablonka et al, 2006). Analysis of the observed phenotype revealed that while *smn-1* silencing caused a gradual disappearance of both ALMs and PLMs, PLMs neurons exhibited a less pronounced reduction compared with ALMs neurons at day 4 of adulthood (Fig 2C). In AVM and PVM neuron, we observed severe defects in the appearance these neurons, with these neurons showing markedly reduced *Pmec-4*::GFP reporter expression (Fig 2C, F and G), or being frequently undetectable. Considering that *mec-4* gene *is* a terminal differentiation marker (Zheng et al, 2018), strong reduction of *Pmec-4*::GFP expression could indicate that *smn-1* silencing is severely impairing proper AVM and PVM differentiation. These neurons sometime appear beyond their normal development timing, consistent with a delayed development. This is followed by a progressive decline over time, and they are largely absent by day 4 of adult worms (Fig 2C). These findings indicate that in *smn-1* silenced animals AVM, PVM, and VD neurons exhibit a delayed development and are possibly more susceptible to degeneration.

### Heterogeneous and cell-specific vulnerability across neuronal populations

This study highlighted several instances of differential vulnerability between neurons of the same class, revealing heterogeneous vulnerability across neuronal populations, where all examined motor and sensory neurons ultimately undergo delay development and degeneration but with varying degrees of sensitivity. This suggests that neuronal susceptibility is not strictly determined by cell class identity but may instead depend on intrinsic neuronal properties, functional state, and developmental context. Consistent with findings reported by Menduti et al (2026), where disruption of inhibitory system function in SMA is associated with altered network stability and selective neuronal vulnerability, our data further support the idea that neuronal maintenance depends on cell-specific factors, including developmental timing, beyond broad neuronal identity. Our observation that VD, AVM, and PVM display both developmental delay and a high propensity for degeneration is consistent with previous findings from human and animal studies, indicating that impaired neuronal development predisposes cells to future degeneration in SMA (Crawford & Pardo, 1996). In addition, studies in zebrafish, fruit fly, and mouse models have reported SMN depletion causing defects to neurogenesis, axonal development, and neuromuscular junction (NMJ) maturation, indicating that there are multiple effects converging on the pathology (McGovern et al, 2008; Martínez-Hernández et al, 2013; Kong et al, 2021). However, due to the greater biological complexity at the cellular and molecular levels of higher order models like mouse, the underlying mechanisms remain difficult to fully elucidate. Our observation of distinct responses to *smn-1* perturbation in individual *C. elegans* neurons provides a foundation for investigating the molecular mechanisms involved in differential sensitivity, ultimately contributing to a better understanding of neurodegenerative disease.

### Structural changes in neuronal process

We also detected progressive structural changes in ALM and PLM neuronal processes, such as shortening and the emergence of branched neurites (Fig 3 G–L). Another notable phenotype observed is ectopic appearance of a posterior neurite in some ALM neurons (Fig 3G–I), a similar phenotype observed in mutants for *mec-7* (Kirszenblat et al, 2013) and *ptrn-1* (Chuang et al, 2014) genes, both being essential for microtubule dynamics. Evidence for defective microtubule stabilization and polymerization has been reported in SMA models and is attributed to the upregulation of Stathmin, a known microtubule-destabilizing factor (Wen et al, 2010). In general, neuronal process branching and ALM posterior neurite appearance observed in our model therefore suggest a defect of microtubule integrity in TRNs, leading to neuronal process abnormalities reminiscent of those observed in other microtubule-defective mutants.

We also identified the presence of swelling all along the neurite of some TRNs when *smn-1* is depleted in our time-lapse analysis, within the period from mid-L2 to 48 h (Fig 3G, H, J, and L). These focal axonal swellings increase in size and number over time*.* This type of axonal swelling has previously been reported in both human and animal SMA models (Coërs and Woolf, 1959) according to McGovern et al (2008) and other neurodegenerative conditions, such as spastic paraplegia and has been attributed to impaired axonal transport (Tarrade et al, 2006). Given that one of the SMN protein’s known functions is in axonal transport (Singh et al, 2017), it is plausible that disruptions in axonal transport or even microtubule integrity might contribute to the appearance of these swellings. These observations highlight the value of our model, as studying such phenomena in the simple and tractable system of *C. elegans* can help clarify mechanisms underlying more complex and controversial findings reported in higher order models. Accordingly, these examples provide a strong foundation for future studies aimed at elucidating the precise mechanisms driving these patterns and their contribution to SMA progression.

### Quantitative analysis of reporter expression

In the course if this work, we paid careful attention to quantitative variations of fluorescent reporter expression. This led to observe neuron-specific quantitative difference in the expression level of *Pflp-13::*GFP which highlights subtle difference in gene expression between neurons from the same class. This is especially relevant since the response to RNAi (also driven by a cell-specific promoter) shows a distinct patterning of sensitivity between individual DD neurons: while *Pflp-13::*GFP expression decreases along the anteroposterior axis, the RNAi sensitivity is more pronounced in DD5 and DD6, then DD1, DD4, and finally DD2 and DD3. What we called sensitivity to RNAi in this case was the proportion of expected neurons that did not produce a visible fluorescent signal in the RNAi strain. It is important to not directly interpret this lack of visibility with absence of the neuron in question. For example, in Fig 1B the green arrow points to a DD3 neuron that could have been considered “absent” for lack of red fluorescent signal if we did not use a 2-color reporter. In this case, the sole presence of the GFP indicates a failure of this neuron to express its full complement of marker genes upon RNAi. When we mention developmental delay in this context, it therefore encompasses the possibility for the neurons in question being present but failing to express the expected markers yet. However, in Gallotta et al (2016), we confirmed that the disappearance of cytoplasmic fluorescent markers in targeted cells was indeed caused by neuronal death of MNs as revealed by positive reactivity to genetic and chemical cell-death markers.

Taken together, these results indicate that the observed changes in reporter expression can sometime reflect an altered neuronal state, including impaired development or differentiation state, rather than simple loss of cells. The cell-specific differences in sensitivity we observed may be caused by distinct RNAi expression levels or other intrinsic differences between these cells. While we did not identify the molecular causes in this work, the stereotypical responses we observe will provide a good model to investigate differential sensitivity to SMN in the future.

Importantly, given that SMN’s primary role is in RNA metabolism, *smn-1* silencing is expected to cause widespread transcriptional disruption. Therefore, the observed defect in reporter expression may reflect an altered transcriptional profile potentially affecting terminal differentiation, or neuronal identity (Bäumer et al, 2009; Corti et al, 2012; Lotti et al, 2012).

In conclusion, we believe our model will be a unique tool for studying the molecular mechanisms of SMN protein function and mechanisms that offer resistance to some neurons in selective neurodegenerative disease like ALS and SMA. Identifying these mechanisms may help determine whether they are conserved in higher order organisms, including mammals, and provide new insights into MN disease.

## Materials and Methods

### *C. elegans* strains and culture conditions

Worms were grown at 20°C onto NGM plates seeded with bacteria (*Escherichia coli*, OP50) as previously described (Brenner, 1974). To maintain the population, worms were transferred onto new plates every 3 d. WT animals were *C. elegans* variety Bristol, strain N2. The alleles and transgenic strains used in this work are summarized in Table S1.

Table S1. Strains used in this study.

### Worm synchronization

Worms were cultured for 2–3 d on seeded NGM plates to obtain gravid adults. Gravid adults were bleached in a solution of sodium hypochlorite (commercial bleach) and NaOH (final concentrations: NaOH 1 M, sodium hypochlorite 0.4–1.6%) and vortexed for 5 min. Once most of the worms had dissolved, bleaching was stopped by adding M9 buffer. The eggs were then pelleted and washed three times with M9 buffer to remove any residual bleach. Finally, the eggs were distributed onto seeded NGM plates and incubated at 20°C to allow further development.

### Neuron-specific RNAi knock-down

To knock-down *smn-1* specifically in TRNs, we used a short form of *mec-3* promoter (417 bp) (Toms et al, 2001) to drive the exon-rich region of *smn-1* used for the RNAi feeding library (Kamath et al, 2003). Exon-rich regions were amplified in two separate PCR reactions to obtain the sense and antisense fragments that were fused to the *mec-3* promoter by PCR fusion using internal primers, as previously described (Hobert 2002; Esposito et al, 2007).

### Transgenic lines

Germline transformation was performed as described previously (Mello et al, 1991). Sense and antisense fusions to the TRNs promoter were pooled in equimolar amounts and microinjected in the N2 strain at 50 ng/μl concentration, to obtain *gbEx518* transgene: *GBF308 mec-3Sp::smn-1 (RNAi sense/antisense); odr-1p::RFP*. The co-injection marker used in this work was: *odr-1p::RFP* (kindly provided by C. Bargmann, The Rockfeller University, New York, USA; RFP expression in AWC and AWB neurons). The injection marker was co-injected at 30 ng/μl.

### Integration of extrachromosomal arrays by ultraviolet irradiation

The injected DNA molecules rearrange to form multicopy extrachromosomal arrays, which can be unstable or mosaic. Integration of these extrachromosomal arrays into the genome provides a means to achieve stable and uniform expression in the progeny, typically through irradiation with ultraviolet or gamma rays (Mariol 2013). In this study, we used ultraviolet irradiation to integrate one of the extrachromosomal arrays, *gbEx518a* transgene, carried by strain NA1164 to generate the stable integrated transgenic strain DUD2202, with the genotype *dudIs2202* [*mec-3Sp::smn-1 *(RNAi sense/antisense); *odr-1p::*RFP] III. To identify the chromosome into which the extrachromosomal array was integrated, we employed *C. elegans* mapping strains.

The integration procedure was performed following the protocol described by Mariol (2013) with minor modifications. Worms carrying an extrachromosomal array with an estimated transmission rate of 20–30% were used. Fifteen L4 or young adult hermaphrodites were transferred onto 10 cm NGM plates seeded with *E. coli* OP50. The plates were irradiated using a UV crosslinker (UV 701435; Jeulin) equipped with a 254 nm UV lamp positioned 5 cm above the plate surface. Worms were exposed to UV light at an intensity of 0.012 J/cm^2^ for 100 s with the plate lids removed. After irradiation, plates were incubated at 20°C to allow the worms to recover and lay eggs. When the progeny reached the young adult stage, fluorescent co-injection markers were used to identify transgenic individuals. Ninety fluorescent F_1_ animals were isolated and transferred individually onto separate 60 mm NGM plates seeded with *E. coli* OP50. These single F_1_ worms were incubated at 20°C until their progeny reached the young adult stage to allow for observation of fluorescent F_2_ animals. Plates showing no progeny or a transmission rate below 30% were discarded. From each selected F_1_ plate, four highly fluorescent transgenic F_2_ animals were isolated and transferred individually onto new 60 mm NGM plates. The resulting F_2_ progeny were subsequently screened for 100% fluorescent F_3_ worms, indicative of successful integration of the transgene.

### Genetic crosses

Hermaphrodite and male worms were transferred to a fresh NGM plate at a ratio of 5:2, along with a drop of *E. coli* OP50 to promote proximity and facilitate mating. A successful cross was identified by the presence of the paternal fluorescent marker in the F_1_ progeny. Four F_1_ worms exhibiting both paternal and maternal fluorescent markers were isolated and allowed to produce F_2_ progeny. From each F_1_ plate, eight F_2_ worms were transferred individually onto separate NGM plates, and their progeny were examined for the presence of 100% expression of both paternal and maternal fluorescent markers, confirming successful establishment of double transgenic lines.

### Behavioral assays

Worms were synchronized by allowing gravid adults to lay eggs for 2–3 h on 6 cm NGM plates seeded with *E. coli* OP50, after which adults were removed. The eggs were then cultured at the appropriate temperature until they reached the young adult stage. For the locomotory response assay, worms were placed on NGM plates seeded with OP50. Gentle touch stimulation was applied using an eyelash hair. Anterior touch (to head region, behind the pharynx) was used to assess backward movement, while posterior touch (to tail region) was used to assess forward movement. This assay was performed on both the *smn-1* silenced strain and the control strain.

For the light-touch (gentle touch) assay, worms were tested on unseeded NGM plates. Light mechanical stimulation was applied by delivering five gentle touches to the anterior region (to head region, behind the pharynx) and five gentle touches to the posterior region (to tail region) using an eyelash hair. This assay was performed on both the *smn-1* silenced strain and the control strain. The number of responses elicited by each stimulus was recorded, and the average response rate was calculated to quantify mechanosensory responsiveness (Goodman, 2006; Nekimken et al, 2017).

### Microscopy observations

Before starting the observations, a 2% agarose pad was prepared on a glass microscope slide. A drop of 10 mmol/L levamisole or sodium azide was applied to the pad to immobilize the worms without causing lethality. Worms were then transferred into the anesthetic solution, and a coverslip was gently placed on top. Observations were performed using a Zeiss Axio Imager Z1 microscope equipped with an HXP 120 lamp and an Axiocam MRM camera for image acquisition. Worms were examined using 10× or 40× objectives under DIC (differential interference contrast) optics, followed by fluorescence imaging (excitation at 395 nm for GFP and 558 nm for dsRed). Neurons were counted manually, and each transgenic strain was analyzed independently rather than through direct comparison of phenotypic outcomes between the two neuronal populations because the promoters used to drive RNAi expression in D-type MNs (*Punc-25* short) and TRNs (*Pmec-3* short) differ, and the extent of *smn-1* silencing may therefore vary between neuronal subtypes.

Long-term imaging experiments were performed as described in Berger et al (2021, 2025). Briefly animals were loaded into the stage appropriate microfluidic long-term imaging device and continuously fed with *E. coli* NA22. Animals were imaged at 15 min intervals for up to 48 h. All images were acquired using an epifluorescence microscope, consisting of a sCMOS camera, an LED light source for fluorescence and bright-field illumination and a piezo objective drive for Z-motorization, using a 40×/1.25NA oil immersion lens. Image acquisition and actuation of the on-chip hydraulic valve was coordinated using a custom MATLAB script. All images were acquired at 20 ± 0.5°C, with temperature control achieved either via the room air-conditioning system, or a microscope cage incubator. Large numbers of animals were imaged as described in Spiri et al (2022), with animals grown on NGM plates until the desired stage is reached. At this point, animals are washed off the NGM plate and loaded into a stage appropriate imaging device and anesthetized using levamisole. Animals were then imaged using an epifluorescence microscope, likewise equipped with a sCMOS camera, an LED light source for fluorescence and bright-field illumination and a piezo objective drive for Z-motorization. Images were acquired using a 40×/1.1NA water immersion lens, and acquisition was coordinated using a custom MATLAB script.

### Quantification of fluorescence intensity

For quantification of fluorescence intensity, we measured the average intensity of pixels within the D-type MN and TRN neuron. The pixel intensity ranges from 0 to 65,535 (Arbitrary unit) in 16-bit images.

For the D-type MNs, we first generated composite images for each worm in FIJI to distinguish VD and DD neurons. This produced three composite images per worm, with each composite correctly representing the intensity of one reporter or the bright-field image. To quantify fluorescent intensity for each reporter in VD or DD neurons, measurements were performed on composite images in which the corresponding reporter channel was set as the primary channel. For TRNs, the creation of composite images was omitted, and intensity measurements were taken directly from the TRN reporter channel. Fluorescence intensity measurements were performed using *Fiji/ImageJ* (version 2.16.0/1.54p). Neurons were first visualized using standard baseline display settings, which restored the display intensity range to its default state based on the actual pixel values in each image. For each neuron, the z-stack slice exhibiting the best focus was selected. An elliptical region of interest (ROI) that best fit the cell body was then manually delineated. For weakly fluorescent neurons that were not detectable under these baseline settings, the display intensity range (i.e., the minimum and maximum values used for image rendering) was adjusted to facilitate identification of the cell body. These adjustments were applied solely for ROI delineation and did not alter the underlying pixel intensity values used for subsequent quantitative analysis. Then, the average intensity of the pixels was calculated for each ROI and was then reported in the Results table and used for subsequent analyses.

### Statistical analysis

Statistical analyses were performed using GraphPad Prism 10 and Python (v3.10.19; SciPy and pandas). In all experiments, independent worms were analyzed at each time point. Data normality was assessed using the Shapiro–Wilk test, and datasets with *P* ≥ 0.05 were considered normally distributed. Neuronal intensity distributions and neurite length distributions were compared pairwise between conditions using the non-parametric Mann–Whitney U test. Backward locomotion test analyzed pairwise between conditions using Fisher’s exact test.

## Supplementary Material

Reviewer comments

## Data Availability

The source data supporting this study are provided. Raw image data for the long-term analysis are available as TIFF (.tif) files. For the high-throughput analysis, processed data are provided in Excel files, and the original TIFF files corresponding to the figures are included. Additional original TIFF files from the high-throughput experiments are available from the corresponding author upon request.
