# Supplementary material for: Individual C. elegans neurons display differential sensitivity to smn-1 silencing
Source: Life Sci Alliance. 2026 Jul 13;9(9):e202603647. doi: 10.26508/lsa.202603647 (PMC13365247; doi:10.26508/lsa.202603647)
Supplement: Reviewer comments [file LSA-2026-03647_review_history.pdf]

Individual C. elegans neurons display differential sensitivity to smn-1 silencing

Sara Savaheli, Ivan Gallotta, Pamela Santonicola, Federica Cieri, Simon Berger, Delphine Dargere, Elia Di Schiavi, and Denis Dupuy
DOI: https://doi.org/10.26508/lsa.202603647

Corresponding author(s): Denis Dupuy, Acides nucléiques: Régulations naturelles et artificielles and Elia Di Schiavi, National Research Council

**Review Timeline:**		
	Submission Date:	2026-01-28
	Editorial Decision:	2026-03-06
	Revision Received:	2026-05-15
	Editorial Decision:	2026-06-15
	Revision Received:	2026-06-22
	Accepted:	2026-06-23

Scientific Editor: Tim Fessenden

Transaction Report:

(Note: With the exception of the correction of typographical or spelling errors that could be a source of ambiguity, letters and reports are not edited. The original formatting of letters and referee reports may not be reflected in this compilation.)

March 6, 2026

Re: Life Science Alliance manuscript #LSA-2026-03647-T

Denis Dupuy

Dear Dr. Dupuy,

Thank you for submitting your manuscript entitled "A Spinal Muscular Atrophy Model Reveals Both Developmental and Degenerative Neuronal Defects in C. elegans" to Life Science Alliance. The manuscript was assessed by expert reviewers, whose comments are appended to this letter.

As you will see, reviewers diverged in their enthusiasm for this work. We appreciate the encouragement from Reviewers 2 and 3, while noting also that Reviewer 2 has concurred with the points raised by Reviewer 1. We invite you to submit a revised manuscript addressing the main concerns raised by Reviewers 1 and 2. Namely, a revised manuscript should include justification for the fluorescent intensity quantification, with validation by an orthogonal method, and characterize which neurons fail to develop in mutants, per Reviewer 1. In addition Reviewer 2 sought confirmation (in the manner of your choice) that loss of smn-1 renders defects on splicing/cell viability. We also agree with text changes and clarifications sought by both reviewers.

I would be happy to discuss the revision in more detail via email or phone/videoconferencing. Please let me know which option you prefer, if any.

To upload the revised version of your manuscript, please log in to your account: https://lsa.msubmit.net/cgi-bin/main.plex

You will be guided to complete the submission of your revised manuscript and to fill in all necessary information. Please get in touch in case you do not know or remember your login name.

The typical timeframe for revisions is three months. Please note that papers are generally considered through only one revision cycle, so strong support from the referees on the revised version is needed for acceptance.

While you are revising your manuscript, please also attend to the below editorial points to help expedite the publication of your manuscript. Please direct any editorial questions to the journal office. When submitting the revision, please include a letter addressing the reviewers' comments point by point.

Thank you for this interesting contribution to Life Science Alliance. We hope that the comments below will prove constructive as your work progresses, and we are looking forward to receiving your revised manuscript.

Sincerely,

Tim Fessenden
Scientific Editor
Life Science Alliance

A. THESE ITEMS ARE REQUIRED FOR REVISIONS

-- A letter addressing the reviewers' comments point by point.

-- An editable version of the final text (.DOC or .DOCX) is needed for copyediting (no PDFs).

-- High-resolution figure, supplementary figure and video files uploaded as individual files: See our detailed guidelines for preparing your production-ready images, https://www.life-science-alliance.org/authors

-- Summary blurb (enter in submission system): A short text summarizing in a single sentence the study (max. 200 characters including spaces). This text is used in conjunction with the titles of papers, hence should be informative and complementary to the title and running title. It should describe the context and significance of the findings for a general readership; it should be written in the present tense and refer to the work in the third person. Author names should not be mentioned.

-- By submitting a revision, you attest that you are aware of our payment policies found here: https://www.life-science-

alliance.org/copyright-license-fee

B. MANUSCRIPT ORGANIZATION AND FORMATTING:

Full guidelines are available on our Instructions for Authors page, https://www.life-science-alliance.org/authors

We encourage our authors to provide original source data, particularly uncropped/-processed electrophoretic blots and spreadsheets for the main figures of the manuscript. If you would like to add source data, we would welcome one PDF/Excel-file per figure for this information. These files will be linked as supplementary "Source Data" files.

IMPORTANT: It is Life Science Alliance policy that if requested, original data images must be made available. Failure to provide original images upon request will result in unavoidable delays in publication. Please ensure that you have access to all original microscopy and blot data images before submitting your revision.

Reviewer #1 (Comments to the Authors (Required)):

In this manuscript, Savaheli et al. attempt to define developmental defects of motor neurons in smn-1 knockdown conditions. The primary weakness of the manuscript is an overreliance on fluorescence reporters to distinguish the presence or absence of neurons, which the authors themselves contradict their own conclusions stating in some sections that absence of signal can conclude that there is a complete lack of neurons, while in other sections they argue that absence of signal alone cannot define this as expression may be altered. This issue is further exacerbated by low resolution imaging and non-traditional quantification methods of already flawed image acquisition. The writing also has some proof-reading issues and many premature conclusions are made from data that do not support such strong statements. The manuscript is also largely descriptive with no attempt at trying to determine what the physiological consequences are of these fluorescent reporters changing in smn-1 knockdown. Overall, in its current state, the manuscript reads as a preliminary study that needs intensive follow-up studies using alternative methods to confirm that the conclusions are valid and that there are physiological ramifications for these changes.
Major issues
-Quantification of the 100 brightest pixels within each individual neuron is not standard for quantification of microscopy images. Selecting only the brightest pixels biases the measurement toward peak signal and excludes a majority of the cell area, making the metric highly sensitive to local hotspots, noise, or saturation effects rather than overall protein abundance or distribution. The cut off of 100 pixels also appears quite arbitrary - where did this number come from? The authors should instead use a more conventional ROI-based metric (such as integrated intensity per neuron). At minimum, the authors should justify the rationale for this measurement and demonstrate that the data are robust using this measurement without any skewing or biasing data.
-Why is there heterogeneity in which specific neurons are gone in the smn-1 RNAi (Fig. 1B)? Is there any scientific rationale for this? This should be quantified further since only 2 images are shown - please make a supplementary figure or table that shows for each animal, which specific neuron is gone and in the discussion please discuss where this stochasticity is coming from. Is it biologically relevant?
-The statement "We noted that the mean fluorescence value observed for the visible neurons in the RNAi individuals was slightly higher than in the controls. This suggests that the undetectable neurons are more likely to be completely missing than present with a strongly diminished expression of the reporter" is not accurate. Please remove this statement. There is no evidence that the neurons are completely missing because the signal cannot be detected. There are so many reasons why the signal can be gone: promoter is turned off, signal is below detection limit, etc. Just because the visible neurons are brighter, this does not allow conclusion that the non-detectable neurons are completely absent - these are unrelated events. This statement is even more questionable when the authors later make a completely contradictory argument that "that ttr-39-promoter-driven mCherry expression might be affected by reductions in SMN-1 levels. This observation reinforces the need for caution when interpreting the absence of a fluorescent signal: the lack of visible expression may indicate the failure of the targeted cell to express its fluorescent marker rather than the absence of the expected neuron." This inconsistency is baffling just paragraphs apart to discuss two contrasting events.
-The conclusion that "smn-1-silenced animals displayed a marked reduction that persisted through adulthood" is an inaccurate statement as measurements were only made until day 1 of adulthood. In order to make this comment, more timepoints needed to be added throughout adulthood. What if smn-1 knockdown delays development? Moreover, the time-lapse imaging is only performed until L2 and claim that some VS neurons fail to appear - L2 is not nearly enough time to make this claim. There is no way to determine whether this is a true developmental failure vs. a developmental delay.
Minor issues
-There are some grammatical errors that need to be fixed; please proof-read. For example, on page 5, it should read "We measured the average intensity of the 100 brightest pixels within each individual neuron", the "of" should be removed. Also, "both control and smn-1-silenced strains" is repeated before the Figure 1E reference. There are many similar instances of these grammatical and writing errors. Please proof-read document carefully.
-Gene nomenclature is wrong in some places. For example, on page 5, it should read "smn-1 RNAi" after the citation of Fig. 1C.

Referee Cross-Comments
-I agree with reviewer #2's comments that greater discussion of utility of the model and validation of the RNAis should be

included.

Reviewer #2 (Comments to the Authors (Required)):

C. elegans has widely been used to model human diseases. Spinal muscular atrophy (SMA) is a devastating disease, and any effort to better understand mechanisms of disease, or to screen for potential therapies, is of wide interest to the field and also to society.
In this manuscript, the authors model the disease in a novel way, depleting smn-1, probably partially, by performing RNAi in particular neuronal lineages in which they observe the phenotype.
It is a valuable contribution to the field, but a better explanation of the utility of this model, compared to other models and deletions of alleles causing mild phenotypes, needs to be discussed... ideally, it should be tested.
Tissue-specific RNAi would have handicaps, including reproducibility or the importance of the timing of depleting mRNAs by RNAi to avoid variability, which need to be avoided for applications like drug screen.
I think a minimal validation at least is requiered to demonstrate that the impact of their siRNAs against smn-1 work similar to other smn-1 models, such us those using mutant alleles. As an example, smn-1 neuronal-type specific affects any of the splicing events described in Rashid et al 2026?...or causes apoptosis, as the same group demonstrated in Gallota et al 2016...in other words, what happens when the signal is gone...also considering that reporters are integrated arrays..(it will be optimal the tagging of endogenous genes to avoid potential silencing).
The deeper discussion about the advantages of this system to model SMA in C. elegans should comment on the option of using degron systems, highly efficient and regulable in C. elegans, rather than RNAi.

Referee Cross-Comments: Overall, I agree with reviewer 1

Reviewer #3 (Comments to the Authors (Required)):

1- the authors targeted the silencing of Smn1 in C elegans in either motor neurone or touch receptor neurone, providing a new model to investigate SMA.
2- a few minor details:
-Could replace "are born" by "emerge" in the 2nd paragraph of the intro.
-Gene names (e.g. SMN1) should be in italic.

In the following text, we indicated the reviewers comments in Blue, our responses in Black and the corresponding changes to the manuscript in Red:

Reviewer #1 (Comments to the Authors (Required)):

The manuscript is also largely descriptive with no attempt at trying to determine what the physiological consequences are of these fluorescent reporters changing in *smn-1* knockdown.

Response:

To address this reviewers's comment we analysed backward movement and responses to light touch in control and RNAi animals. We observed that both are impaired following *smn-1* silencing consistent with functional deficiency of the corresponding neurons. These results are presented in the new panel C in Figure 1 and E-F in Figure 3.

Corresponding text modification:

Results:
"We also confirmed a significant reduction of backward locomotion following light touch behind the pharynx in RNAi animal compared to control (Figure 1C, as previously described in (Gallotta et al., 2016))."

Results:
"We assessed functional changes resulting from neuronal process defects by performing a light-touch assay in controls and *smn-1*–silenced worms. We observed a significant reduction of light touch response in young adult animals in *smn-1*–silenced worms, which was more pronounced in posterior touch, consistent with the more severe neuronal process defects observed in PLMs (Figure 3E, F) (Nekimken et al. 2017)."

Methods:
"**Behavioral assays**
Worms were synchronized by allowing gravid adults to lay eggs for 2–3 hours on 6 cm NGM plates seeded with E. coli OP50, after which adults were removed. The eggs were then cultured at the appropriate temperature until they reached the young adult stage. For the locomotory response assay, worms were placed on NGM plates seeded with OP50. Gentle touch stimulation was applied using an eyelash. Anterior touch (to head region, behind the pharynx) was used to assess backward movement, while posterior touch (to tail region) was used to assess forward movement. This assay was performed on both the *smn-1* silenced strain and the control strain.
For the light touch (gentle touch) assay, worms were tested on unseeded NGM plates. Light mechanical stimulation was applied by delivering five gentle touches to the anterior region (to head region, behind the pharynx) and five gentle touches to the posterior region (to tail region) using an eyelash hair. This assay was performed on both the *smn-1* silenced strain and the control

strain. The number of responses elicited by each stimulus was recorded, and the average response rate was calculated to quantify mechanosensory responsiveness (Nekimken et al. 2017; Goodman 2006) ."

Major issues

-Quantification of the 100 brightest pixels within each individual neuron is not standard for quantification of microscopy images. Selecting only the brightest pixels biases the measurement toward peak signal and excludes a majority of the cell area, making the metric highly sensitive to local hotspots, noise, or saturation effects rather than overall protein abundance or distribution. The cut off of 100 pixels also appears quite arbitrary - where did this number come from? The authors should instead use a more conventional ROI-based metric (such as integrated intensity per neuron). At minimum, the authors should justify the rationale for this measurement and demonstrate that the data are robust using this measurement without any skewing or biasing data.

Response: We followed this reviewer recommendation and used a standard ROI based quantification method to evaluate the fluorescent intensity of the neurons of interest.
The new figure panels 1E, 2D, 2E, 2F, and 2G are now based on this quantification. The corresponding section of the Methods has also been modified accordingly. Taking into account all the pixels in the ROI leads to similar observations as were previously made using only the most expressed pixels.

-Why is there heterogeneity in which specific neurons are gone in the smn-1 RNAi (Fig. 1B)? Is there any scientific rationale for this?

Response: We thank the reviewer for this comment, as we believe this is the most interesting finding in this work: we observed that individual neurons had consistently different expression level for their respective reporters across individual animals. For example, in DD neurons there is an anteroposterior gradient *Pflp-13::GFP* intensity observable both in control and RNAi worms (Figure 1E), while the level of expression of *Pttr-39::mCherry* is mostly identical across all 6 DD neurons. In control TRNs, we also observe a difference between AVM and PVM expression level of *Pmec-4::GFP* at the L2 stage, indicating a different kinetic of gene expression during development.

These observations were described in more detail in the modified text:

Results: "Measuring the fluorescence intensity revealed that individual neurons had consistently different fluorescence level across individual animals. For example, in DD neurons there is an anteroposterior gradient for *Pflp-13* driven GFP-intensity observable both in control and *smn-1*–silenced worms (Figure 1E, upper panel). By contrast, in *Pttr-39*::mCherry expressing animals, the reporter levels appeared relatively uniform across DD neurons, with no pronounced intra-

subclass differences (Figure 1E, lower panel). Overall, the fluorescence levels of both reporters are not significantly affected by *smn-1* silencing compared to controls (Figure 1E)."

Results: "GFP intensity was markedly lower in *smn-1* silenced worms than in the control in all TRNs. This reduction became more pronounced over time and was accompanied by a progressive decrease in the number of detectable neurons (Figure 2D-G)."
Discussion: "This led to observe neuron-specific quantitative difference in the expression level of *Pflp-13::*GFP which highlights subtle difference in gene expression between neurons from the same class. This is especially relevant since the response to RNAi (also driven by a cell-specific promoter) shows a distinct patterning of sensitivity between individual DD neurons: while *Pflp-13::*GFP expression decreases along the anteroposterior axis, the RNAi sensitivity is more pronounced in DD5 and DD6, then DD1, DD4 and finally DD2 and DD3. What we called sensitivity to RNAi in this case was the proportion of expected neurons that did not produce a visible fluorescent signal in the RNAi strain."
Discussion: "The cell specific differences in sensitivity we observed may be caused by distinct RNAi expression levels or other intrinsic differences between these cells. While we did not identify the molecular causes in this work, the stereotypical responses we observe will provide a good model to investigate differential sensitivity to SMN in the future."

This should be quantified further since only 2 images are shown - please make a supplementary figure or table that shows for each animal, which specific neuron is gone and in the discussion please discuss where this stochasticity is coming from. Is it biologically relevant?

We believe these individual differences to be biologically relevant since we observed different sensitivity to RNAi between individual neurons of the same class. We added figure panels 1D and 2C to illustrate this.
In figure 1D, we show that DD5 and DD6 are the most sensitive to *smn-1* gene perturbation followed by DD1 and DD4, DD2 and DD3 being the most resilient. These sensitivity level do not correlate with the *flp-13* reporter expression level suggesting additional differences between these neurons.
The supplementary figures S3-6 presents a larger sampling of control and RNAi animal images.

In figure 2C, we can see that the PLM is the most resilient of the TRNs (as far as D4 adults) while the AVM and PVM that developed later than in the control also disappear as early as L4, with PVM showing a faster disappearance rate.

These observations were described in more detail in the modified text:

Results: "While all 6 DD neurons were always visible in the control strain, we observed neuron-specific depletion in the RNA-interfered strain. DD5 and DD6 were the most sensitive to *smn-1* gene perturbation, with only 1-2 animal out of 10 displaying detectable fluorescence in these neurons. DD1 was the next most affected neuron, visible in only 4 out of ten animals. DD2 and DD3 were the most resilient ones, detected in 8 to 9 RNAi animals out of ten, respectively (Figure

1D). In addition, we observed several DD neurons that only express GFP instead of both expected fluorescent reporters (green arrowhead in Figure 1B and green cells in Figure 1D; Supplementary Video 1)."

Results: "By the day 4 of adult stage, the proportion of visible neurons decreased to 0.15 in the ALMs and 0.45 in the PLMs (Figure 2C). For post-embryonically born neurons (AVM and PVM), in about half of the animals we did not detect either or both AVM and PVM by the L2 stage. From L2 to L4 stage the fraction of visible AVMs went from 0.64 to 0.8, potentially indicating a developmental delay. For PVM in contrast, this fraction went from 0.72 to 0.58. By day 4 of adult stage the ratio of missing neurons rose to 0.86 in AVM and 0.98 in PVM (Figure 2C)."

We added the following text to the discussion:
Discussion: "We found that, VD neurons exhibit a more pronounced progressive loss compared to DD neurons (Figure 1G). Together, these findings indicate distinct vulnerability profiles among motor neuron subtypes and between individual cells with those subtypes."

Discussion: "Analysis of the observed phenotype revealed that while *smn-1* silencing caused a gradual disappearance of both ALMs and PLMs, PLMs neurons exhibited a less pronounced reduction compared to ALMs neurons at day 4 of adulthood (Figure 2C). In AVM and PVM neuron, we observed severe defects in the appearance these neurons, with these neurons showing markedly reduced *Pmec-4*::GFP reporter expression (Figure 2C, F-G), or being frequently undetectable. Considering that *mec-4* gene *is* a terminal differentiation marker (Zheng et al. 2018), strong reduction of *Pmec-4*::GFP expression could indicate that *smn-1* depletion is severely impairing proper AVM and PVM differentiation. These neurons sometime appear beyond their normal development timing, consistent with a delayed development. This is followed by a progressive decline over time, and they are largely absent by day 4 of adult worms (Figure 1C). These findings indicate that in *smn-1* silenced animals AVM, PVM andVD neurons exhibit a delayed development and are possibly more susceptible to degeneration."

And because we feel that this is the strongest point of this manuscript, we propose to change the title to:
"Individual *C. elegans* neurons display differential sensitivity to *smn-1* silencing"

-The statement "We noted that the mean fluorescence value observed for the visible neurons in the RNAi individuals was slightly higher than in the controls. This suggests that the undetectable neurons are more likely to be completely missing than present with a strongly diminished expression of the reporter" is not accurate. Please remove this statement.

Response : We removed this statement that went indeed further than what we intended.

There is no evidence that the neurons are completely missing because the signal cannot be detected. There are so many reasons why the signal can be gone: promoter is turned off, signal is below detection limit, etc. Just because the visible neurons are brighter, this does not allow conclusion that the non-detectable neurons are completely absent - these are unrelated events. This statement is even more questionable when the authors later make a completely contradictory argument that "that *ttr-39*-promoter-driven mCherry expression might be affected by reductions in SMN-1 levels. This observation reinforces the need for caution when interpreting the absence of a fluorescent signal: the lack of visible expression may indicate the failure of the targeted cell to express its fluorescent marker rather than the absence of the expected neuron." This inconsistency is baffling just paragraphs apart to discuss two contrasting events.

Response: We agree with this comment, we removed both statements from the results and added a few sentences in the discussion to clarify this important topic:

Discussion: "It is important to not directly interpret this lack of visibility with absence of the neuron in question. For example, in Figure 1B the green arrow points to a DD3 neuron that could have been considered "absent" for lack of red fluorescent signal if we didn't use a 2-color reporter. In this case the sole presence of the GFP indicates a failure of this neuron to express its full complement of marker genes upon RNAi. When we mention developmental delay in this context, it therefore encompasses the possibility for the the neurons in question being present but failing to express the expected markers yet. However, in (Gallotta et al. 2016) we confirmed that the disappearance of cytoplasmic fluorescent markers in targeted cells was indeed caused by neuronal death of MNs as revealed by positive reactivity to genetic and chemical cell-death markers.
Taken together, these results indicate that the observed changes in reporter expression can sometime reflect an altered neuronal state, including impaired development or differentiation state, rather than simple loss of cells."
Discussion p15: "Importantly, given that SMN's primary role is in RNA metabolism, *smn-1* silencing is expected to cause widespread transcriptional disruption. Therefore, the observed defect in reporter expression may reflect an altered transcriptional profile potentially affecting terminal differentiation, or neuronal identity (Corti et al. 2012; Lotti et al. 2012; Bäumer et al. 2009)."

-The conclusion that "*smn-1*-silenced animals displayed a marked reduction that persisted through adulthood" is an inaccurate statement as measurements were only made until day 1 of adulthood. In order to make this comment, more timepoints needed to be added throughout adulthood. What if *smn-1* knockdown delays development? Moreover, the time-lapse imaging is only performed until L2 and claim that some VS neurons fail to appear - L2 is not nearly enough time to make this claim. There is no way to determine whether this is a true developmental failure vs. a developmental delay.

Response: We added the day 4 adult time point to figure 1F-G, 2C

Minor issues

-There are some grammatical errors that need to be fixed; please proof-read. For example, on page 5, it should read "We measured the average intensity of the 100 brightest pixels within each individual neuron", the "of" should be removed. Also, "both control and *smn-1*-silenced strains" is repeated before the Figure 1E reference. There are many similar instances of these grammatical and writing errors. Please proof-read document carefully.

-Gene nomenclature is wrong in some places. For example, on page 5, it should read "*smn-1* RNAi" after the citation of Fig. 1C.

Response:

We carefully edited the new version of the manuscript to fix remaining errors.

Referee Cross-Comments

-I agree with reviewer #2's comments that greater discussion of utility of the model and validation of the RNAis should be included.

Response: We added the following section of the discussion that includes other models benefits and caveats.

Discussion: "Although several SMA models have been developed in *C. elegans*, the study of SMA at later developmental stages remains limited due to the larval lethality of complete *smn-1* knockout (*smn-1*(*ok355*) mutant) and of systemic RNAi of *smn-1*. In addition, hypomorphic models (e.g. *smn-1*(*cb131*) and *smn-1*(*gk118916*) mutants) do not exhibit obvious neuronal loss at larval and adult stages, limiting their ability to recapitulate the neurodegenerative features observed in SMA(Briese et al. 2009; Miguel-Aliaga et al. 1999; Doyle et al. 2020). Interestingly, *smn-1*(*gk118916*) show D-type MNs degeneration at 9 days of adulthood, but, being a genetic mutant, it does not allow to assess the cell-autonomous role played by *smn-1* in specific subclasses of neurons. To overcome this, we used a model in which *smn-1* is silenced in defined neuronal populations using neuron-specific RNAi approach (Esposito et al. 2007)."

Reviewer #2 (Comments to the Authors (Required)):

I think a minimal validation at least is requiered to demonstrate that the impact of their siRNAs against *smn-1* work similar to other *smn-1* models, such us those using mutant alleles. As an example, smn-1 neuronal-type specific affects any of the splicing events described in Rashid et al 2026?… or causes apoptosis, as the same group demonstrated in Gallota et al 2016...in other words, what happens when the signal is gone...also considering that reporters are integrated arrays..(it will be optimal the tagging of endogenous genes to avoid potential silencing).

Response:

We thank this reviewer for bringing this paper to our attention. As the modifications of splicing observed in that study only affect about 10% of reads at the organism level we have no possibility to assess this effect in the few cells that are affected in our model. This caveat is now addressed in the modified discussion section mentioned above.

For the apoptosis question: since we previously demonstrated that was the effect of our RNAi approach (Gallota et al 2016) we assumed it was still valid as we are using the same strategy.

Response:

The deeper discussion about the advantages of this system to model SMA in C. elegans should comment on the option of using degron systems, highly efficient and regulable in C. elegans, rather than RNAi.

Response: We modified the discussion to include the following paragraph:

Discussion: "Although several SMA models have been developed in *C. elegans*, the study of SMA at later developmental stages remains limited due to the larval lethality of complete *smn-1* knockout (*smn-1*(*ok355*) mutant) and of systemic RNAi of *smn-1*. In addition, hypomorphic models (e.g. *smn-1*(*cb131*) and *smn-1*(*gk118916*) mutants) do not exhibit obvious neuronal loss at larval and adult stages, limiting their ability to recapitulate the neurodegenerative features observed in SMA(Briese et al. 2009; Miguel-Aliaga et al. 1999; Doyle et al. 2020). Interestingly, *smn-1*(*gk118916*) show D-type MNs degeneration at 9 days of adulthood, but, being a genetic mutant, it does not allow to assess the cell-autonomous role played by *smn-1* in specific subclasses of neurons. To overcome this, we used a model in which *smn-1* is silenced in defined neuronal populations using neuron-specific RNAi approach (Esposito et al. 2007). Thus, worms remain viable and healthy, and neurons develop with normal levels of SMN until *smn-1* silencing onset once the promoter driving RNAi expression becomes active. Using this strategy, earlier studies have shown that *smn-1* silencing in *C. elegans* GABAergic MNs significantly compromise the survival of neurons in an age-dependent manner (Gallotta et al. 2016)."
"

Reviewer #3 (Comments to the Authors (Required)):

1- the authors targeted the silencing of Smn1 in C elegans in either motor neuron or touch receptor neuron, providing a new model to investigate SMA.
2- a few minor details:

-Could replace "are born" by "emerge" in the 2nd paragraph of the intro.
Response: We did as suggested.

-Gene names (e.g. SMN1) should be in italic.
Response: We corrected the nomenclature throughout the manuscript.

June 15, 2026

RE: Life Science Alliance Manuscript #LSA-2026-03647-TR

Dr. Denis Dupuy
Acides nucléiques: Régulations naturelles et artificielles
2 rue Dr Hoffmann-Martinot
ARNA
Bordeaux 33000
France

Dear Dr. Dupuy,

Thank you for submitting your revised manuscript entitled "Individual C. elegans neurons display differential sensitivity to smn-1 silencing". As you will see, both reviewers are satisfied with no further requests. We would be happy to publish your paper in Life Science Alliance pending final revisions necessary to meet our formatting guidelines.

MANUSCRIPT ORGANIZATION AND FORMATTING:

To avoid unnecessary delays in the acceptance and publication of your paper, please read the following information carefully. Full guidelines are available on our Instructions for Authors page, https://www.life-science-alliance.org/authors

Submission of a paper that does not conform to Life Science Alliance guidelines will delay the acceptance of your manuscript.

-Please add your main, supplementary figure, table, and video legends to the main manuscript text after the references section and remove the separate file containing the supplementary legends. Please upload the Supplementary Table as an individual file.
-Please add a Summary Blurb/Alternate Abstract and a Category in our system.
-Please add the X and Bluesky handles of your host institute/organization, as well as your own, and/or one of the authors, in our system.
-The titles in both the system and the manuscript file must be consistent with each other. Please update the title in the system so it matches the manuscript file.
-Please rename "Ethics declarations" to "Conflict of Interest" and "Bibliography" to "References."
-Please reorder supplementary figure legends so that you start with figure S1, S2, S3, S4, S5, and S6.
-Please place a "Data Availability" section after the Materials & Methods section. Please consult our guidelines at https://www.life-science-alliance.org/manuscript-prep#format
-Please add callouts for Figures 3K; S2; S3; S4; S5 and S6 to your main manuscript text.

We encourage our authors to provide original source data, particularly uncropped/-processed electrophoretic blots and spreadsheets for the main figures of the manuscript. If you would like to add source data, we would welcome one PDF/Excel-file per figure for this information. These files will be linked as supplementary "Source Data" files.

It is Life Science Alliance policy that if requested, original data images must be made available to the editors. Failure to provide original images upon request will result in unavoidable delays in publication. Please ensure that you have access to all original data images prior to final submission.

If you are planning a press release on your work, please inform us immediately to allow informing our production team and scheduling a release date.

We welcome submissions of potential cover images for the issue of LSA in which your work would appear. If you have high quality images associated with this work, please feel free to email these, with a caption, to the journal office.

LSA encourages authors to provide a 30-60 second video where the study is briefly explained. These videos will be appear embedded with the manuscript online at Life Science Alliance, and on social media to promote the published paper and authors (for examples, see https://docs.google.com/document/d/1-UWCfbE4pGcDdcgzcmiuJl2XMBJnxKYeqRvLLrLSo8s/edit?usp=sharing). Corresponding or first-authors are welcome to submit the video. Please submit only one video per manuscript. The video can be emailed to contact@life-science-alliance.org

FINAL FILES:

To upload the final version of your manuscript, please log in to your account: https://lsa.msubmit.net/cgi-bin/main.plex
You will be guided to complete the submission of your revised manuscript and to fill in all necessary information. Please get in touch in case you do not know or remember your login name.

The following items are required for acceptance.

-- An editable version of the final text (.DOC or .DOCX) is needed for copyediting (no PDFs).

-- High-resolution figure, supplementary figure and video files uploaded as individual files: See our detailed guidelines for preparing your production-ready images, https://www.life-science-alliance.org/authors

-- Summary blurb (enter in submission system): A short text summarizing in a single sentence the study (max. 200 characters including spaces). This text is used in conjunction with the titles of papers, hence should be informative and complementary to the title. It should describe the context and significance of the findings for a general readership; it should be written in the present tense and refer to the work in the third person. Author names should not be mentioned.

The license to publish form must be signed before your manuscript can be sent to production. A link to the license to publish form will be available to the corresponding author only. Please take a moment to check your funder requirements.

Reviews, decision letters, and point-by-point responses associated with peer-review at Life Science Alliance will be published, alongside the manuscript. If you do want to opt out of having the reviewer reports and your point-by-point responses displayed, please let us know immediately.

Thank you for your attention to these final processing requirements. Please revise and format the manuscript and upload materials as soon as you are able.

Thank you for this interesting contribution to the literature. We look forward to publishing your paper in Life Science Alliance.

Sincerely,

Tim Fessenden
Scientific Editor
Life Science Alliance

Reviewer #1 (Comments to the Authors (Required)):

The authors have gone above and beyond in responding to all reviewer critiques and the new data provided have significantly strengthened the manuscript. I have no further comments and believe the revised version of the manuscript is very strong and suitable for publication.

Reviewer #2 (Comments to the Authors (Required)):

Although authors did not address the experimental requests from my first review, they have provided a reasonable response to my comments and I do not have major issues or problems in the publication of this manuscript.

June 23, 2026

RE: Life Science Alliance Manuscript #LSA-2026-03647-TRR

Dr. Denis Dupuy
Acides nucléiques: Régulations naturelles et artificielles
2 rue Dr Hoffmann-Martinot
ARNA
Bordeaux 33000
France

Dear Dr. Dupuy,

Thank you for submitting your Research Article entitled "Individual C. elegans neurons display differential sensitivity to smn-1 silencing". It is a pleasure to let you know that your manuscript is now accepted for publication in Life Science Alliance. Congratulations on this interesting work.

The final published version of your manuscript will be deposited by us to PubMed Central upon publication.

Your manuscript will now progress through copyediting and proofing. It is journal policy that authors provide original data upon request.

Your article will publish open access upon publication under a CC-BY license.

Reviews, decision letters, and point-by-point responses associated with peer-review at Life Science Alliance will be published, alongside the manuscript. If you do want to opt out of having the reviewer reports and your point-by-point responses displayed, please let us know immediately.

IMPORTANT: If you will be unreachable at any time, please provide us with the email address of an alternate author. Failure to respond to routine queries may lead to unavoidable delays in publication.

Scheduling details will be available from our production department. You will receive proofs shortly before the publication date. Only essential corrections can be made at the proof stage so if there are any minor final changes you wish to make to the manuscript, please let the journal office know now.

DISTRIBUTION OF MATERIALS:
Authors are required to distribute freely any materials used in experiments published in Life Science Alliance. Authors are encouraged to deposit materials used in their studies to the appropriate repositories for distribution to researchers.

You can contact the journal office with any questions, contact@life-science-alliance.org

Again, congratulations on a very nice paper. I hope you found the review process to be constructive and are pleased with how the manuscript was handled editorially. We look forward to future exciting submissions from your lab.

Sincerely,

Tim Fessenden
Scientific Editor
Life Science Alliance
